# Validation of the third version of the Copenhagen Psychosocial Questionnaire for Portugal

**DOI:** 10.1371/journal.pgph.0006036

**Published:** 2026-05-19

**Authors:** Teresa P. Cotrim, Pedro Bem-Haja, Paula Vagos, Isabel Silva, Rui Azevedo, Cláudia Fernandes, Samuel Antunes, Joaquim S. Pinto, Elisabeth Brito, Isabel Souto, Iara Teixeira, Carlos F. Silva, Anabela Pereira

**Affiliations:** 1 Ergonomics Laboratory, Faculdade de Motricidade Humana, Universidade de Lisboa‌‌, Lisbon, Portugal; 2 CIAUD, Centro de Investigação em Arquitetura, Urbanismo e Design, Faculdade de Arquitetura, Universidade de Lisboa, Lisbon, Portugal; 3 CINTESIS, Department of Education and Psychology, University of Aveiro, Aveiro‌‌, Portugal; 4 WJCR, Department of Education and Psychology, University of Aveiro, Aveiro, Portugal; 5 Escola de Psicologia, Universidade do Minho, Braga, Portugal; 6 CICS.NOVA.UMinho, Universidade do Minho, Braga, Portugal; 7 University of Maia (UMAIA), Maia, Portugal; 8 Center ALGORITMI, University of Minho, Braga, Portugal; 9 NECE-UBI, Research Center for Business Sciences, University of Beira Interior, Covilhã, Portugal; 10 CATIM, Technological Center, Porto, Portugal; 11 Departamento de Psicologia, Universidade Autónoma de Lisboa, Lisbon, Portugal; 12 IETA, Dep.to de Eletrónica, Telecomunicações e Informática, University of Aveiro, Aveiro, Portugal; 13 GOVCOPP, School of Technology and Management of Águeda, University of Aveiro, Aveiro, Portugal; 14 CIDTFF, Department of Education and Psychology, University of Aveiro, Aveiro, Portugal; 15 Department of Psychology, University of Évora, Research Center on Education and Psychology (CIEP), Évora, Portugal; Islamic Azad University South Tehran Branch, IRAN, ISLAMIC REPUBLIC OF

## Abstract

The prevalence and impact of exposure to psychosocial risk factors seems to be increasing in labour contexts in general. The assessment and measurement of psychosocial work factors is essential for the improvement of healthy workplaces and should be incorporated into health and safety management programmes. The assessment of psychosocial factors requires the selection of appropriate instruments that respond to the multidimensionality of that construct. The COPSOQ III covers a multitude of theoretical approaches and provides comprehensive information on psychosocial working conditions that may help to deduce actions for their improvement. The use of COPSOQ III in national context needs prior validation studies. In this regard, the paper aims to assess the psychometric properties of the middle version of COPSOQ III as to provide evidence on the validity of this instrument to be used for the Portuguese working population. The validation process comprised a national sample of 7,506 participants that included different occupational sectors based on a cross-sectional study. Construct validity was assessed through Confirmatory Factor Analysis and the internal consistency of the scales was evaluated using Cronbach’s alpha. The results revealed a satisfactory reliability and ability of the Portuguese middle version of COPSOQ III (COPSOQ III-PT) to discriminate the psychosocial dimensions. The distribution of the scores of the study sample by each scale and by sex enables a better interpretation of the results based on reference values.

## Background

Psychosocial factors have gained enormous relevance in the last two decades due to social and technological changes and to global threats that impact the labour sectors of society, such as the recent COVID-19 pandemic. The current relevance of psychosocial factors in work contexts and the positive or negative impact that exposure to them has on working populations in terms of their physical, mental and social health and of the sustainability of employment is unquestionable, and is related to the goals of achieving decent work for all [1,2]. Although it is not consensual, one of the most adopted definitions of psychosocial factors mentions that they cover aspects of work design, management, and related to the social and organizational contexts that have the potential to cause psychological or physical harm [3].

The prevalence and impact of exposure to psychosocial risk factors seems to be increasing in labour contexts in general. This phenomenon is due not only to increased knowledge of this problem and its assessment, but, to a large extent, to changes in work organization models [4]. Work systems are moving towards an ever-increasing introduction of technologies and the advent of artificial intelligence is creating new challenges in the management of psychosocial risks. The presence of artificial intelligence, robotics and machine learning systems is increasing in sectors such as health, industry, finance, education and others, with consequences for the physical and mental health of workers [5]. Among the most common risk factors resulting from the interaction of workers with new technological systems in organizations are workload, time pressure, loss of autonomy, weak social relationships and job insecurity, which lead to stress, dissatisfaction, a poor work-life balance, among other consequences for the health and well-being of these workers [5].

The assessment and measurement of psychosocial work factors is recognized as essential for the development of healthy workplaces [6–9] and should be incorporated into health and safety management programmes [5]. Organizations need to have policies and strategies that respond to the demands of a regulatory and legal system framed by occupational health and safety. This requires the implementation of collective risk assessment measures, for psychosocial risks and all others, which will in turn allow for the definition of risk mitigation and control measures, or of health promoting measures [10].

When assessing psychosocial factors, one of the key issues is selecting the most appropriate and psychometrically sound instruments that respond to the multidimensionality of that construct. In practice, that means access to tools and techniques for gathering adequate information about aspects associated with psychosocial factors. On the other hand, in Europe, the business structure is mostly made up of micro, small and medium-sized enterprises, where risk assessment is generally more difficult, which also happens with psychosocial risks [11]. In this context, there is a clear need to provide employers and those responsible for health and safety management in organizations with robust tools to identify psychosocial factors and whether they constitute health promoters or risk factors.

To deal with the scarcity of tools available to identify the psychosocial factors and to respond to the inclusion of different concepts and dimensions that are proposed to be part of that construct, an international group was established to oversee the implementation and validation of the Copenhagen Psychosocial Questionnaire (COPSOQ) starting in the year 2000 [12,13]. Over time, this group evolved into the current steering committee, which continuously evaluates the theory and practice underlying the application of COPSOQ [14]. Thanks to their efforts, versions I and II of the COPSOQ have been revised and updated to its latest version III [12,15]. The COPSOQ III covers a multitude of theoretical approaches and provides comprehensive information on psychosocial working conditions that may help to deduce actions for their improvement [16–18].

Due to the need for researchers and technicians to have a comprehensive tool to assess psychosocial factors, the COPSOQ rapidly became a reference questionnaire with multiple adaptations and validation processes in numerous countries. The use of COPSOQ III in any national context needs prior validation studies, to make it suitable for research and application in various organizational settings [15]. The psychometric properties of the COPSOQ III have been extensively studied, demonstrating its validity, reliability, applicability and relevance in several cultural contexts [6,15,16,19–26].

In Portugal, the COPSOQ questionnaire is one of the most widely used instruments in organizations, intervention contexts and research due to its multidimensionality and applicability [27–30]. The COPSOQs’ validation process began in 2006 and resulted in the validation of the middle version of the COPSOQ II in 2011, which allowed the creation of reference values for each scale by sex-based, age group, and sector of activity [31]. In parallel with the international development of the COPSOQ II into the COPSOQ III, the process of validating the middle version of the COPSOQ III began, resulting in the publication of its preliminary version that ensured the contents and face validity of the items and performed field-testing to reduce the number of items and obtain insight into the data structure [16]. This preliminary middle version of COPSOQ III proved to be a valid version for future validation studies with various populations. However, an in-depth validation study of the Portuguese preliminary middle version of COPSOQ III referring to a wide sample of the working population was necessary to assess the robustness and the psychometric properties of the questionnaire and, in a near future, establish reference values.

The current paper aims to assess the psychometric properties of the middle version of COPSOQ III as to provide evidence on the validity and reliability of this instrument to be used for the Portuguese working population and contribute to a good management of psychosocial factors in the workplace. In this regard, the validation process included evaluating construct validity and internal consistency. Considering previous preliminary findings using the Portuguese middle version of the tool [16], adequate psychometric properties are expected.

## Materials and methods‌‌

The study was cross-sectional and based on the application of the COPSOQ III questionnaire, through the online platform of the Portuguese Observatory of Occupational Psychosocial Factors (https://fatorespsicossociais.pt).

### Participants

The validation of the middle version of COPSOQ III for Portugal (COPSOQ III-PT) comprised a national sample that included different occupational sectors based on a cross-sectional study. Data collection was carried out at the level of Portuguese companies. The companies and their employees, who participated in this study were recruited from various occupational sectors across the different regions of mainland Portugal, including public and private organizations. The sampling process was stratified by activity sector, with different sectors having different proportions of the sample.

Cross-sectional data collection occurred from the 1^st^ of March 2021 to the 30^th^ of November 2023. In the companies that consented to the study, data was collected by applying the preliminary Portuguese middle version of the COPSOQ III questionnaire [16]. Data were collected via the online platform of the Portuguese Observatory of Occupational Psychosocial Factors that enforced item completion. Participants could only submit the questionnaire once they had responded to all items. Consequently, there were no missing values at item level for the COPSOQ III scales.

The final sample comprised 7,506 participants.

### Ethical procedures

The study was approved by the Ethics Committee of the Faculty of Human Kinetics (number 9/2021), and the Ethics Committee for Research in Social and Human Sciences of University of Minho (CEICSH 012/2023). The organizations were invited to participate in the study regarding the coverage of different regions of the country and diverse activity sectors. Those that agreed to take part in the study distributed the link to the questionnaire, based on the platform of the Portuguese Observatory of Occupational Psychosocial Factors (https://fatorespsicossociais.pt), to all employees through internal channels. Participation was, therefore, anonymous and confidential. Before filling in the online questionnaire, workers had to accept the informed consent and agree that their participation was voluntary and anonymous. Participants were required to read the informed consent information, after which they were asked to indicate in writing whether they accepted participation in the study. Only after agreeing to participate did they have access to the online questionnaire. The group-based psychosocial profile was sent back to each organization for internal distribution, along with the definition of risk prevention and health promotion measures.

### Instrument

The preliminary Portuguese Middle Version of the COPSOQ III (COPSOQ III-PT) was used in the present study, which emerged from the initial validation study (see S1 File). It encompasses 85 items distributed by 31 factors that can be aggregated in seven dimensions. The Portuguese middle version of COPSOQ III introduced three new factors, “Control over Working Time”, “Quality of Work”, and “Insecurity over Working Conditions” when compared with the Portuguese middle version of COPSOQ II [16], as recommended by the COPSOQ Steering Committee. The Portuguese version adhered to the guidelines of maintaining the core items for all scales [15]. This means that a total of 32 core items were identified, along with an additional 24 items from the middle version and 25 items from the long version [16] (Table 1). Additionally, socio-demographic data regarding sex, age, occupation, and activity sector was also collected.

**Table 1:** Psychosocial dimensions, factors, number of items, and question numbers of the preliminary Portuguese middle version of COPSOQ III (adapted from Cotrim et al. [16]).

Psychosocial Dimension	Factors	Nº of items	Question numbers
Demands at Work	Quantitative Demands (QD)	3	Q01; Q02; Q03
	Work Pace (WP)	2	Q04; Q05
	Cognitive Demands (CD)	4	Q06; Q07; Q08; Q09
	Emotional Demands (ED)	3	Q10; Q11; Q12
Work Organization and Job Contents	Influence at work (IN)	4	Q13; Q14; Q15; Q16
	Development Possibilities (PD)	3	Q17; Q18; Q19
	Control Over Working Time (CT)	3	Q20; Q21; Q22
	Meaning of Work (MW)	3	Q23; Q24; Q25
Interpersonal Relations and Leadership	Predictability (PR)	2	Q29; Q30
	Recognition (RE)	3	Q31; Q32; Q33
	Role Clarity (CL)	3	Q34; Q35; Q36
	Role Conflicts (CO)	3	Q37; Q38; Q39
	Quality of Leadership (QL)	4	Q40; Q41; Q42; Q43
	Social Support from Colleagues (SC)	3	Q44; Q45; Q46
	Social Support from Supervisors (SS)	3	Q47; Q48; Q49
	Sense of Community at Work (SW)	3	Q50; Q51; Q52
Social Capital	Horizontal Trust (HT)	3	Q59; Q60; Q61
	Vertical Trust (VT)	3	Q62; Q63; Q64
	Organizational Justice (JU)	4	Q65; Q66; Q67; Q68
Work-Individual Interface	Commitment to the workplace (CW)	3	Q26; Q27; Q28
	Job Insecurity (JI)	2	Q53; Q54
	Insecurity over Working Conditions (IW)	3	Q55; Q56; Q57
	Quality of work (QW)	1	Q58
	Work-Life Conflict (WF)	3	Q69; Q70; Q71
	Job Satisfaction (JS)	3	Q72; Q73; Q74
Personality	Self-Efficacy (SE)	2	Q76; Q77
Health and Well-Being	Self-Rated Health (GH)	1	Q75
	Sleeping Troubles (SL)	2	Q78; Q79
	Burnout (BO)	2	Q80; Q81
	Stress (ST)	2	Q82; Q83
	Depressive Symptoms (DS)	2	Q84; Q85

### Statistical analysis

The calculation of all the scales was based on the average of the items and each item varies between 1 and 5 points. The higher value can be either favorable or unfavorable depending on the direction of the scale.

To assess the construct validity of the 31-factor solution of the Portuguese middle version of COPSOQ III, a Confirmatory Factor Analysis (CFA) using the weighted least squares mean and variance adjusted estimator (WLSMV) was conducted. This estimator was used for its robustness in the presence of violations of multivariate normality. In confirmatory factor analysis (CFA), different indices were used to capture complementary aspects of model fit. The Comparative Fit Index (CFI) and Tucker–Lewis Index (TLI) compared the fit of the hypothesized model to a null model; values above 0.90 are generally considered acceptable, and values above 0.95 indicate very good fit. The Root Mean Square Error of Approximation (RMSEA) estimated the discrepancy between the model and the population covariance matrix per degree of freedom; values below 0.07 indicate acceptable fit, and values below 0.05 reflect a close fit. The Standardized Root Mean Square Residual (SRMR) represents the standardized difference between observed and predicted correlations, with values below 0.08 generally indicating good fit [32]. Local Adjustment was evaluated through the calculation of the lambdas (factorial loads), and by verifying if they exceeded the 0.5 cut-off proposed by Hair et al. [32].

Discriminant validity of the constructs within our structural equation model was assessed using the Heterotrait-Monotrait ratio (HTMT) of correlations, as advocated by Hair et al. [32]. Mathematically, the HTMT reflects the average of all cross-construct indicator correlations (heterotrait-heteromethod correlations), normalized by the geometric mean of the within-construct indicator correlations (monotrait-heteromethod correlations). This ratio provides a relative measure, assessing whether the shared variance between distinct constructs is sufficiently low to affirm discriminant validity. The HTMT is a contemporary criterion for establishing the discrimination of factor constructs, wherein a threshold value below 0.90 indicates sufficient discriminant validity.

The internal consistency of the scales in our study was evaluated using Cronbach’s alpha, with higher values indicating greater reliability of the scale, and values above 0.7 generally considered acceptable for psychological constructs [33]. For each scale, several statistical measures were reported: mean, median, standard deviation, range, and the 10th through 100th percentiles at 10% intervals.

To assess the robustness of our confirmatory factor analysis model across sex-based groups, an invariance analysis was conducted. This analysis was crucial to determine whether the underlying construct measurement models were equivalent for male and female participants, allowing for meaningful comparisons across these groups. The invariance analysis was structured in a hierarchical manner, progressing through three levels of invariance testing: configural, metric, and scalar.

All analyses were performed using R.

## Results

The current study presents the results regarding construct validity and reliability of the Portuguese Middle version of COPSOQ III in a large sample of workers.

### Socio-demographic characteristics

The study sample comprised a total of 7,506 participants, distributed across various professional sectors. The sex distribution within the sample was as follows: 57.4% identified as female (n = 4,308), 27.0% as male (n = 2,030), 0.1% as other (n = 11), and 0.2% preferred not to disclose their sex (n = 14). Additionally, 15.2% of the participants’ sex-based data were not collected due to compliance to companies internal requirements regarding anonymization of data (n = 1,143). Age could not be analyzed in the present study, although it was collected in most organizations that took part in the study, because age groups were not homogeneous due to restrictions related to the combination of data protection regulations and company preferences.

In terms of sector distribution, the participants were spread across several fields: Public Administration (Central Administration: 6.5%, n = 486; Local Administration: 8.8%, n = 658), Agriculture (0.4%, n = 28), Commerce (0.5%, n = 40), Education (11.8%, n = 888), Industry (24.6%, n = 1,850), Health (11.2%, n = 837), Services (28.6%, n = 2,144), and others (7.7%, n = 575).

### Construct validity

Mardia’s test showed that data was not multivariate normal: χSkew = 323344.768, p < 0.001; ZKurtosis = 531.307, p < 0.001. Given this result, Confirmatory Factor Analysis using weighted least-square-mean and variance adjusted estimator (WLSMV) was conducted. Results of the 31-factor model of COPSOQ III revealed an acceptable global adjustment: robust CFI = 0.971; robust TLI = 0.966; RMSEA = 0.041, RMSEA 90% CI [0.040, 0.041]; SRMR = 0.043. However, the mathematical assumptions required to confer good local adjustment to the scale were not met, as item 27 from the “Commitment to the Workplace” (CW) reached a factor weight of 0.1. Such a minimal factor loading suggests a weak association with its intended underlying construct, raising concerns about the item’s contribution to the overall scale. Furthermore, the individual reliability associated with this item, indicated by a value above 0.05 [32], hinting at a non-negligible level of measurement error, thereby questioning its utility and consistency within the scale. Recognizing the potential detriment posed by the inclusion of item 27 and considering that this item is not part of the core items defined by the original authors of the scale [15], a decision was made to omit this item from the “Commitment to the Workplace” scale.

The revised model demonstrated very good global fit indices, indicating that the hypothesized structure is strongly supported by the data. The Comparative Fit Index (CFI = 0.977) and Tucker–Lewis Index (TLI = 0.972) were both well above the commonly accepted threshold of 0.90 and close to 1, suggesting that the specified model explains the observed covariances much better than a null or independence model. The Root Mean Square Error of Approximation (RMSEA = 0.038, 90% CI [0.037, 0.039]) further confirmed the adequacy of the model, since values below 0.05 are generally interpreted as evidence of a close fit, and even values below 0.07 are considered acceptable. This narrow confidence interval reinforces the stability of the estimate and reduces concerns about potential model misfit. Finally, the Standardized Root Mean Square Residual (SRMR = 0.039) was well below the 0.08 cutoff, showing that the discrepancies between observed and predicted correlations are minimal.

When interpreted together, these indices converge to demonstrate that the Portuguese middle version of COPSOQ III exhibits excellent structural validity. The model not only meets but exceeds conventional fit standards, indicating that the factorial structure provides a reliable and accurate representation of psychosocial constructs in the context of a large and heterogeneous national sample.

Post-removal, all remaining items exhibited substantial factor loadings (λ > 0.45), indicating a strong and relevant connection to their respective latent constructs [32]. The meticulous exclusion of the underperforming item fortified the 31-factor solution’ construct validity, which now exhibits good local and global fit in this version (Table 2).

**Table 2:** Confirmatory factor analysis (CFA) results for 31-factor solution of the COPSOQ III Portuguese middle version using WLSMV estimation.

Factor	Item	Estimate	SE	Z	p	Standardized Estimate
Quantitative Demands (QD)	Q01	1.000				0.921
	Q02	0.710	0.019	36.648	0.000	0.669
	Q03	0.739	0.019	38.073	0.000	0.697
Work Pace (WP)	Q04	1.000				0.856
	Q05	0.937	0.019	50.087	0.000	0.814
Cognitive Demands (CD)	Q06	1.000				0.451
	Q07	1.471	0.053	27.857	0.000	0.583
	Q08	1.376	0.064	21.408	0.000	0.494
	Q09	2.197	0.091	24.242	0.000	0.773
Emotional Demands (ED)	Q10	1.000				0.925
	Q11	0.763	0.017	45.770	0.000	0.639
	Q12	0.939	0.015	64.526	0.000	0.828
Influence at Work (IN)	Q13	1.000				0.690
	Q14	0.989	0.033	30.189	0.000	0.658
	Q15	1.185	0.036	32.971	0.000	0.758
	Q16	0.979	0.032	30.358	0.000	0.713
Possibilities for Development (PD)	Q17	1.000				0.744
	Q18	1.046	0.023	45.828	0.000	0.822
	Q19	1.212	0.023	51.744	0.000	0.891
Control over Working Time (CT)	Q20	1.000				0.637
	Q21	1.070	0.039	27.708	0.000	0.694
	Q22	1.063	0.035	30.566	0.000	0.723
Meaning of Work (MW)	Q23	1.000				0.696
	Q24	0.937	0.021	44.210	0.000	0.680
	Q25	1.436	0.032	44.853	0.000	0.929
Commitment to the Workplace (CW)	Q26	1.000				0.588
	Q28	1.482	0.036	40.675	0.000	0.883
Predictability (PR)	Q29	1.000				0.747
	Q30	0.887	0.015	59.264	0.000	0.740
Recognition (RE)	Q31	1.000				0.723
	Q32	1.059	0.016	65.599	0.000	0.792
	Q33	1.051	0.016	65.353	0.000	0.840
Role Clarity (CL)	Q34	1.000				0.863
	Q35	0.664	0.016	41.510	0.000	0.680
	Q36	0.790	0.018	42.946	0.000	0.719
Role Conflicts (CO)	Q37	1.000				0.561
	Q38	1.302	0.043	30.310	0.000	0.782
	Q39	1.361	0.049	27.942	0.000	0.804
Quality of Leadership (QL)	Q40	1.000				0.784
	Q41	0.982	0.015	64.274	0.000	0.794
	Q42	1.047	0.017	62.459	0.000	0.800
	Q43	1.132	0.018	62.072	0.000	0.851
Social Support from Colleagues (SC)	Q44	1.000				0.844
	Q45	1.026	0.017	59.087	0.000	0.843
	Q46	0.722	0.022	32.775	0.000	0.572
Social Support from Supervisors (SS)	Q47	1.000				0.824
	Q48	1.081	0.016	67.281	0.000	0.889
	Q49	0.871	0.014	63.527	0.000	0.727
Sense of Community at Work (SW)	Q50	1.000				0.769
	Q51	1.341	0.023	58.953	0.000	0.880
	Q52	1.140	0.017	66.746	0.000	0.831
Job Insecurity (JI)	Q53	1.000				0.862
	Q54	0.944	0.027	35.403	0.000	0.822
Insecurity over Working Conditions (IW)	Q55	1.000				0.642
	Q56	1.375	0.047	29.510	0.000	0.842
	Q57	1.085	0.044	24.554	0.000	0.651
Quality of Work (QW)	Q58	1.000				0.576
Horizontal Trust (HT)	Q59	1.114	0.035	31.987	0.000	0.613
	Q60*	-1.147	0.040	-28.733	0.000	-0.597
	Q61*	-1.041	0.038	-27.368	0.000	-0.531
Vertical Trust (VT)	Q62	1.000				0.758
	Q63	1.032	0.017	62.124	0.000	0.781
	Q64	1.297	0.022	59.887	0.000	0.821
Organizational Justice (JU)	Q65	1.000				0.819
	Q66	0.964	0.013	75.944	0.000	0.779
	Q67	1.048	0.013	79.890	0.000	0.801
	Q68	1.029	0.015	68.800	0.000	0.785
Work-Life Conflict (WF)	Q69	1.000				0.941
	Q70	0.959	0.010	98.969	0.000	0.880
	Q71	0.992	0.011	90.901	0.000	0.905
Job Satisfaction (JS)	Q72	1.000				0.865
	Q73	0.885	0.011	77.566	0.000	0.850
	Q74	0.924	0.014	66.145	0.000	0.804
Self-Rated Health (GH)	Q75	1.000				1.000
Self-Efficacy (SE)	Q76	1.000				0.534
	Q77	1.190	0.045	26.155	0.000	0.653
Sleeping Troubles (SL)	Q78	1.000				0.856
	Q79	1.016	0.018	56.239	0.000	0.854
Burnout (BO)	Q80	1.000				0.815
	Q81	1.222	0.016	75.319	0.000	0.942
Stress (ST)	Q82	1.000				0.863
	Q83	0.971	0.014	67.896	0.000	0.777
Depressive Symptoms (DS)	Q84	1.000				0.864
	Q85	0.982	0.013	74.807	0.000	0.848

* reverse-scored items.

Regarding Discriminant validity, the HTMT analysis revealed that none of the construct pairs exceeded the critical threshold of 0.90 (the computed HTMT values for all possible pairs of constructs are presented in S2 File). This outcome substantiates the empirical distinctness of the constructs and corroborates the integrity of the measurement scales employed in the study. Consequently, the constructs are inferred to operate as discrete entities, distinctly measured by their respective indicators, devoid of substantial overlap in their conceptual domains.

### Reliability

The scales’ reliability for our measures span from 0.52 to 0.96, signifying an overall satisfactory level of internal consistency (Table 3). Most scales demonstrated acceptable to excellent internal consistency. However, two scales fell below the conventional acceptability criterion: “Self-Efficacy” (SE; α = 0.52) and “Commitment to the Workplace” (CW; α = 0.63). While these values may initially raise concerns about the scales’ reliability, it is important to recognize that Cronbach’s alpha is influenced by both the average inter-item correlation and the number of items, tending to underestimate reliability in short scales. The Self-Efficacy scale comprised only two items and showed low internal consistency (α = 0.52), corresponding to an inter-item correlation of r = 0.351. While the items demonstrate a moderate association, the resulting reliability is limited due to the short scale length.

**Table 3:** Characteristics of the scales and reliability analysis through Cronbach’s alpha.

Scale	Positive value	Nº of Items	Alpha	Floor Effect in %*	Ceiling Effect in %*
Quantitative Demands (QD)	Low	3 items	0.85	4.0	1.0
Work Pace (WP)	Low	2 items	0.82	1.9	8.3
Cognitive Demands (CD)	Low	4 items	0.72	0.2	4.0
Emotional Demands (ED)	Low	3 items	0.87	3.7	3.6
Influence at Work (IN)	High	4 items	0.83	1.1	2.7
Possibilities for Development (PD)	High	3 items	0.89	0.5	15.2
Control over Working Time (CT)	High	3 items	0.78	1.1	7.9
Meaning of Work (MW)	High	3 items	0.87	0.9	14.1
Commitment to the Workplace (CW)	High	2 items	0.63	1.5	9.3
Predictability (PR)	High	2 items	0.71	1.4	4.9
Recognition (RE)	High	3 items	0.86	1.3	8.1
Role Clarity (CL)	High	3 items	0.86	0.4	18.6
Role Conflicts (CO)	Low	3 items	0.79	1.0	2.2
Quality of Leadership (QL)	High	4 items	0.89	1.6	6.9
Social Support from Colleagues (SC)	High	3 items	0.83	0.4	5.5
Social Support from Supervisors (SS)	High	3 items	0.89	2.5	5.0
Sense of Community at work (SW)	High	3 items	0.91	0.7	17.3
Job Insecurity (JI)	Low	2 items	0.83	22.6	8.1
Insecurity over Working Conditions (IW)	Low	3 items	0.80	12.1	4.5
Quality of Work (QW)	High	1 item	–	3.2	15.9
Horizontal Trust (HT)	High	3 items	0.70	0.5	4.6
Vertical Trust (VT)	High	3 items	0.87	0.6	8.9
Organizational Justice (JU)	High	4 items	0.90	1.2	3.8
Work-Life Conflict (WF)	Low	3 items	0.96	7.1	3.4
Job Satisfaction (JS)	High	3 items	0.91	1.6	4.5
Self-Rated Health (GH)	Low	1 item	–	7.4	3.9
Self-Efficacy (SE)	High	2 items	0.52	0.2	6.1
Sleeping Troubles (SL)	Low	2 items	0.84	9.5	3.9
Burnout (BO)	Low	2 items	0.87	5.1	6.0
Stress (ST)	Low	2 items	0.80	5.7	3.7
Depressive Symptoms (DS)	Low	2 items	0.85	10.5	3.0

*Floor and ceiling effects were calculated based on the mean scores of the items comprising each subscale.

### Reference values

Since it was not found full scalar invariance between males and females, a comprehensive descriptive analysis for each of the COPSOQ III scales is provided in Table 4 for females and Table 5 for males. For each subscale, several statistical measures are reported: mean, median, standard deviation, range, and the 10th through 100th percentiles at 10% intervals. These metrics offer an in-depth view of the data distribution, facilitating understanding of the central tendencies and variability associated with each subscale, and allowing Portuguese users of the COPSOQ III to position their own scores relatively.

**Table 4:** Percentile table for each scale of the COPSOQ III, for females.

Scale	Mean	SD	Range	10th	20th	30th	40th	50th	60th	70th	80th	90th	100th
QD	2.72	0.881	4.00	1.67	2.00	2.33	2.33	2.67	3.00	3.00	3.53	4.00	5.00
WP	3.42	0.896	4.00	2.00	2.50	3.00	3.00	3.50	3.50	4.00	4.00	4.50	5.00
CD	3.78	0.647	4.00	3.00	3.25	3.50	3.75	3.75	4.00	4.00	4.25	4.50	5.00
ED	3.20	0.962	4.00	2.00	2.33	2.67	3.00	3.33	3.33	3.67	4.00	4.33	5.00
IN	3.17	0.838	4.00	2.00	2.50	2.75	3.00	3.25	3.50	3.75	4.00	4.25	5.00
PD	3.82	0.856	4.00	2.67	3.00	3.33	3.67	4.00	4.00	4.33	4.67	5.00	5.00
CT	3.44	0.950	4.00	2.00	2.67	3.00	3.33	3.67	3.67	4.00	4.33	4.67	5.00
MW	3.91	0.825	4.00	3.00	3.33	3.67	4.00	4.00	4.00	4.33	4.67	5.00	5.00
CW	3.44	0.887	4.00	2.50	3.00	3.00	3.50	3.50	3.50	4.00	4.00	4.50	5.00
PR	3.18	0.845	4.00	2.00	2.50	3.00	3.00	3.00	3.50	3.50	4.00	4.00	5.00
RE	3.49	0.902	4.00	2.33	2.67	3.00	3.33	3.67	3.67	4.00	4.33	4.67	5.00
CL	4.07	0.742	4.00	3.00	3.67	3.67	4.00	4.00	4.33	4.67	4.67	5.00	5.00
CO	3.02	0.731	4.00	2.00	2.33	2.67	3.00	3.00	3.00	3.33	3.67	4.00	5.00
QL	3.37	0.939	4.00	2.00	2.50	3.00	3.00	3.50	3.75	4.00	4.00	4.50	5.00
SC	3.61	0.793	4.00	2.67	3.00	3.33	3.33	3.67	4.00	4.00	4.33	4.67	5.00
SS	3.12	0.977	4.00	2.00	2.33	2.67	3.00	3.00	3.33	3.67	4.00	4.33	5.00
SW	3.85	0.845	4.00	2.67	3.00	3.33	3.67	4.00	4.00	4.33	4.67	5.00	5.00
JI	2.51	1.25	4.00	1.00	1.00	1.50	2.00	2.50	3.00	3.00	3.50	4.50	5.00
IW	2.68	1.12	4.00	1.00	1.67	2.00	2.33	2.67	3.00	3.33	3.67	4.33	5.00
QW	3.71	0.836	4.00	3.00	3.00	3.00	4.00	4.00	4.00	4.00	4.00	5.00	5.00
HT	3.42	0.780	4.00	2.33	2.67	3.00	3.33	3.33	3.67	4.00	4.00	4.33	5.00
VT	3.63	0.790	4.00	2.67	3.00	3.33	3.67	3.67	4.00	4.00	4.33	4.67	5.00
JU	3.17	0.832	4.00	2.00	2.50	2.75	3.00	3.00	3.25	3.50	4.00	4.25	5.00
WF	2.96	1.01	4.00	1.67	2.00	2.33	2.67	3.00	3.00	3.33	4.00	4.00	5.00
JS	3.33	0.812	4.00	2.33	2.67	3.00	3.00	3.33	3.67	4.00	4.00	4.00	5.00
GH	3.02	0.892	4.00	2.00	2.00	3.00	3.00	3.00	3.00	3.00	4.00	4.00	5.00
SE	3.76	0.595	4.00	3.00	3.50	3.50	3.50	4.00	4.00	4.00	4.00	4.50	5.00
SL	2.85	1.04	4.00	1.50	2.00	2.00	2.50	3.00	3.00	3.50	4.00	4.00	5.00
BO	3.23	0.963	4.00	2.00	2.50	3.00	3.00	3.00	3.50	4.00	4.00	4.50	5.00
ST	2.99	0.938	4.00	2.00	2.00	2.50	3.00	3.00	3.00	3.50	4.00	4.00	5.00
DS	2.69	0.991	4.00	1.50	2.00	2.00	2.50	2.50	3.00	3.00	3.50	4.00	5.00

**Table 5:** Percentile table for each scale of the COPSOQ III, for males.

Scale	Mean	SD	Range	10th	20th	30th	40th	50th	60th	70th	80th	90th	100th
QD	2.66	0.882	4.00	1.67	2.00	2.00	2.33	2.67	3.00	3.00	3.33	4.00	5.00
WP	3.31	0.911	4.00	2.00	2.50	3.00	3.00	3.00	3.50	4.00	4.00	4.50	5.00
CD	3.78	0.657	4.00	3.00	3.25	3.50	3.75	3.75	4.00	4.00	4.25	4.50	5.00
ED	2.92	0.953	4.00	1.67	2.00	2.33	2.67	3.00	3.00	3.33	3.67	4.33	5.00
IN	3.28	0.832	4.00	2.25	2.50	3.00	3.00	3.25	3.50	3.75	4.00	4.25	5.00
PD	3.80	0.863	4.00	2.67	3.00	3.33	3.67	4.00	4.00	4.33	4.67	5.00	5.00
CT	3.59	0.886	4.00	2.33	3.00	3.00	3.33	3.67	4.00	4.00	4.33	4.67	5.00
MW	3.86	0.886	4.00	2.67	3.33	3.67	3.67	4.00	4.00	4.33	4.67	5.00	5.00
CW	3.47	0.929	4.00	2.00	2.50	3.00	3.50	3.50	4.00	4.00	4.50	4.50	5.00
PR	3.26	0.899	4.00	2.00	2.50	3.00	3.00	3.00	3.50	4.00	4.00	4.50	5.00
RE	3.56	0.965	4.00	2.33	2.67	3.00	3.33	3.67	4.00	4.00	4.33	4.67	5.00
CL	4.08	0.795	4.00	3.00	3.33	3.67	4.00	4.00	4.33	4.67	5.00	5.00	5.00
CO	3.03	0.749	4.00	2.00	2.33	2.67	3.00	3.00	3.00	3.33	3.67	4.00	5.00
QL	3.46	0.993	4.00	2.00	2.75	3.00	3.25	3.50	4.00	4.00	4.25	4.75	5.00
SC	3.60	0.783	4.00	2.67	3.00	3.33	3.67	3.67	3.67	4.00	4.33	4.67	5.00
SS	3.25	0.991	4.00	2.00	2.33	2.67	3.00	3.33	3.67	4.00	4.00	4.33	5.00
SW	3.94	0.854	4.00	2.97	3.33	3.67	4.00	4.00	4.00	4.33	4.67	5.00	5.00
JI	2.40	1.220	4.00	1.00	1.00	1.50	2.00	2.00	2.50	3.00	3.50	4.00	5.00
IW	2.64	1.110	4.00	1.00	1.67	2.00	2.33	2.67	3.00	3.33	3.67	4.00	5.00
QW	3.80	0.874	4.00	3.00	3.00	3.00	4.00	4.00	4.00	4.00	4.00	5.00	5.00
HT	3.58	0.798	4.00	3.00	3.00	3.00	3.33	3.67	3.67	4.00	4.33	4.67	5.00
VT	3.63	0.856	4.00	2.33	3.00	3.33	3.33	3.67	4.00	4.00	4.33	4.67	5.00
JU	3.28	0.880	4.00	2.00	2.50	2.75	3.00	3.25	3.50	3.75	4.00	4.50	5.00
WF	2.84	1.01	4.00	1.33	2.00	2.00	2.67	3.00	3.00	3.33	4.00	4.00	5.00
JS	3.38	0.865	4.00	2.33	2.93	3.00	3.00	3.33	3.67	4.00	4.00	4.33	5.00
GH	2.79	0.964	4.00	2.00	2.00	2.00	3.00	3.00	3.00	3.00	4.00	4.00	5.00
SE	3.83	0.624	4.00	3.00	3.50	3.50	3.50	4.00	4.00	4.00	4.50	4.50	5.00
SL	2.59	1.040	4.00	1.00	1.50	2.00	2.00	2.50	3.00	3.00	3.50	4.00	5.00
BO	2.80	1.000	4.00	1.50	2.00	2.00	2.50	3.00	3.00	3.00	3.50	4.00	5.00
ST	2.67	0.968	4.00	1.50	2.00	2.00	2.50	2.50	3.00	3.00	3.50	4.00	5.00
DS	2.42	0.978	4.00	1.00	1.50	2.00	2.00	2.50	2.50	3.00	3.00	4.00	5.00

Regarding women, on the “Cognitive Demands” scale, results were predominantly close to the most unfavourable extreme, with a score of 3 at the 10th percentile, rising gradually to the maximum score of 5 at the 100th percentile. Nevertheless the ceiling effect for this scale was of 4% (Table 4). On the scales of “Role Clarity”, “Quality of Work” and “Self-Efficacy”, the sample was close to the most favourable extreme, scoring 3 at the 10th percentile, 4 at the 40th, 50th and 60th percentiles and 5 at the 90th and 100th percentiles. “Role Clarity” and “Quality of Work” presented ceiling effects of 18.6% and 15.9%, respectively (Table 5). A similar pattern on the same scales was found in males (Table 4 and Table 5).

## Discussion

Given the relevance of the individual, organisational and social impact of exposure to psychosocial risk factors, there is an increasing need for their continuous assessment in work contexts. The conceptualisation of psychosocial factors, particularly psychosocial risk factors, is still not consensual, but there is agreement on their multidimensionality [1,34]. Commonly the psychosocial working conditions are assessed through the experience of the individuals being exposed to those conditions, meaning that for practical reasons, that exposure is frequently measured by self-report instruments [34]. The multidimensionality of this concept implies the need for instruments suited to this characteristic, validated in different countries, and with large applicability.

Regarding the assessment of psychosocial factors, the evolution of COPSOQ and, in particular, the development of COPSOQ III has been based not only on conceptual criteria, but also on the need to compare different populations and samples from different countries, respecting specific needs and cultural differences while maintaining comparability between countries. This has been achieved on the basis of the core-item concept. This concept refers to the set of items that must be maintained in all versions from all countries, as a guarantee of comparability. Each country, in its process of developing and validating national versions, must add, from the middle and long versions, the remaining items to the scales [15]. Bearing this in mind, the validation processes in the different countries are based on a common base regarding the core items, but face a challenging process regarding comparability of the results between countries.

The development of the Portuguese middle version of COPSOQ III respected the fact that Portuguese companies made widespread use of the middle version of COPSOQ II [27]. Based on this conservative perspective and aiming to minimize the risk of hampering comparisons [35], the third Portuguese version was based on the second one. The changes suggested for COPSOQ III, such as new scales and rephrasing some items, were introduced in the new Portuguese version [16].

In this regard, the present study analysed the psychometric characteristics of the Portuguese middle version of COPSOQ III (COPSOQ III-PT), namely construct validity and reliability. Construct validity encompassed the assessment of structural validity using a Confirmatory Factor Analysis, and of the discriminant validity of the constructs by the Heterotrait-Monotrait ratio (HTMT) of correlations. Reliability was examined by the use of the alpha of Cronbach for internal consistency.

The analysis was performed with a sample of Portuguese workers from different sectors of activity, representing 0,4% of the primary sector, 24,6% of the secondary and 75% of the tertiary sector. The Portuguese working population is estimated at 4 426 461 workers, from those, 2,9% work in the primary sector, 24,8% in the secondary and 72,3% in the tertiary [36]. Although our sample is underrepresented in the primary sector, the global distribution is similar to the national one, with a heterogenous composition of the sample regarding occupations and geographical regions. This imbalance is consistent with structural and methodological constraints commonly reported in occupational and psychosocial survey research, as primary-sector workers are more geographically dispersed, predominantly rural, and less accessible through digital recruitment strategies [37,38]. Some references indicate that validation studies should prioritise sample size adequacy and heterogeneity across relevant occupational contexts [39]. In this regard, the overall sectoral distribution of the sample approximates national employment distribution in the secondary and tertiary sectors, including a wide range of occupations and geographical regions. These characteristics support the evaluation of the Portuguese version of COPSOQ III’s measurement properties, though the underrepresentation of the primary sector must be considered when interpreting sector-specific generalisability.

Regarding sex-based distribution among the employed population in Portugal, the sex-based gap was about 5,5% in 2024 [40]. The distribution of workers based on sex in our sample does not follow the same pattern of the Portuguese population, with a higher representation of female respondents and about 15% of data not collected. Non-response to demographic items, such as sex, is common and may be related to perceived sensitivity or respondent burden [41]. Also, the overrepresentation of females is frequent and has happened in other validation studies on COPSOQ III [6,42]. In any case, incomplete sex data are unlikely to affect the evaluation of core measurement properties, when sample heterogeneity is preserved and appropriate missing-data handling is applied [43]. Nevertheless, this limitation should be considered when interpreting sex-specific analyses.

### Validity analysis

The COPSOQ is based on a multidimensional construct, and the instrument reflects this multidimentionality by the use of scores for each scale [12,13] instead of a global score, or a score per dimension.

Construct validity was used to provide evidence of validity [44] and, within this, structural validity is of utmost relevance and was assessed through factor confirmatory analysis. Our results highlighted the need to exclude one more item from the version that had already undergone an item reduction process in the preliminary analysis [16]. After the removal of item 27 from the scale “Commitment to the Workplace”, the model revealed good local and global fit indices, suggesting a robust alignment with the empirical data. This structural validity analysis evidence that the Portuguese middle version of COPSOQ III adequately reflects the multidimensionality of the psychosocial construct. Additionaly, item 27 was eliminated since it was not a core item, in line with the recommendations of the authors who developed versions II and III of COPSOQ. This two-item scale was already included in the short version of COPSOQ II and was only included in the long version of COPSOQ III [15,16]. The Portuguese version will retain this scale as it already existed in the Portuguese middle version of COPSOQ II.

Other studies analysed the structural validity of the instrument by the use of Confirmatory Factor Analysis (CFA), but referring to a different number of factors (mentioned as scales in this study) and items, from 17 in the validation study for the Canadian working population [45], to 25 factors in the Turkish validation study [25], both showing good models fit. The Australian validation studies used the long version of COPSOQ III, which comprised 43 factors. However, after refinement and item exclusion, the final version contained 36 factors and demonstrated a good model fit [42]. The Norwegian validation study performed a similar study with a sample of nurses. After the item reduction process it ended with 86 items and 28 factors, and CFA showed also a good model fit [24]. Similar to the Portuguese study, all these studies carried out CFA based on factors and their respective items. Good results were obtained, indicating that the items meaningfully contribute to their corresponding constructs and reflecting the multidimensionality of a psychosocial assessment tool such as the COPSOQ.

### Reliability analysis

The reliability analysis through Cronbach’s Alpha showed mainly acceptable to very good internal consistency of the scales. Only two scales, both with two items, presented low reliability with values lower than 0.70: the “Self-Efficacy” (SE) scale with 0.52 and “Commitment to the Workplace” (CW) with 0.63. The modest alpha values for “Self-Efficacy” and, to a lesser extent, “Commitment to the Workplace” are likely attributable in part to scale brevity rather than solely to poor item coherence. Nonetheless, the internal consistency of the “Self-Efficacy” scale remains limited, and results involving this measure should be interpreted with caution, particularly when drawing conclusions at the individual level, where higher reliability is typically required [33]. With regard to the Self-Efficacy scale, there is another aspect that may contribute to poorer internal consistency. This is the lower variability of the results, which can be seen in the distribution by percentile for both men and women.

The remaining scales showed alpha values that ranged from 0.70 (“Horizontal Trust”) to 0.96 (“Work-Life Conflict”). It is worth noting that most of the scales vary between two and four items, which influences the results of Cronbach’s alpha [44]. On the other hand, the fact that the sample is very heterogeneous, as it was recruited from different activity sectors, various regions of the country, integrating females and males, different age groups, various levels of education and occupational groups (from elementary occupations until managers, excluding armed forces), may contribute to a higher alpha value, as heterogeneity of the sample will allow higher values of alpha [44]. Globally, our results are in line with those reported in the international validation study [15]: acceptable values of alpha, although the values are slightly higher for most of the scales in the present study; the highest value of alpha was found for the scale “Work-Life Conflict” in both studies (0.84 in the international study, with two items; 0.96 in the Portuguese study, with three items). The scale “Work-Life Conflict” in the Portuguese version has three items, which may explain a higher alpha compared with the international validation study [15]. In the preliminary version of the instrument, the scale “Commitment to the Workplace” included three items. Following the analysis of structural validity, the number of items were reduced to two, which may have influenced the reliability results for this scale. In the international validation study the “Commitment to the Workplace” was also a two item scale, and the reliability results were similar (0.64) to ours (0.63). Based on these reliability findings, the scales “Self-Efficacy” and “Commitment to the Workplace” may require further revision (e.g., adding or refining items). As is, the use of these scales should be done with caution and within the goal of describing psychosocial work factors, but not to characterize or make decisions within workplaces.

### Reference values

The distribution of the scores of the study sample by each scale and by sex enables a better interpretation of the results [44]. The results of the scales’ “Cognitive demands” (CD), “Role Clarity” (CL) and “Quality of Work” (QW) were close to the higher values, which indicates that the sample is clustered at the highest points of these scales. When compared with other european versions, the cognitive demands scale was not included in the Swedish [6], nor in the German versions [21]. Considering that scores clustering at extremes might impact accuracy and lead to biased results, it may be considered a future analysis of this scale. Regarding the “Role Clarity” scale, both European versions showed high ceiling effects 10.6% to 16.1% [6,21]. “Quality of Work” was used in the Swedish version with two items and a ceiling effect of 6.6% [6]. As the number of items may influence these effects, our results may depend on the scale having only one item. The permanence of the scale “Quality of Work” in the instrument should be balanced between its contribution and the burden on participants.

The reference values obtained in this national study enable the results of studies conducted in various occupational contexts to be compared and priorities for practical action to be identified. The Portuguese middle version of COPSOQ III provides researchers and practitioners with an essential tool for identifying, monitoring and managing psychosocial factors at company and national levels. Regularly collecting reliable information is vital for informing local and sectoral policies and strategies in psychosocial risks prevention. It is also worth noting that the Portuguese version of the COPSOQ II is widely recognized and used in organizational and academic contexts. It is even recommended by government bodies in the field of psychosocial risk prevention. In this sense, the validation of the Portuguese version of COPSOQ III represents a cutting-edge development, allowing for an updated and more comprehensive psychosocial assessment in Portugal. Beyond reinforcing international comparability, this new version gives organizations and national authorities with a more robust, and up-to-date tool to support occupational health policies and practices.

### Strengths and limitations

The large size of the sample, with a proportion by activity sector similar to that of the Portuguese labour force, although not fully representative of it, is one of the main advantages of this study. Although our results are not population based, they are based on a large national stratified sample regarding region and activity sector, allowing the development of reference values usefull for companies or researchers. Additionaly, as our sample is underrepresented in the primary sector, the results may not be generalisable to this sector and may introduce limitations in the use of the data. Further studies in this sector will be needed. It should also be noted that due to the fact that the gender distribution in the sample does not reflect that of the Portuguese population and 15% of the data is missing, the results should be interpreted with caution.

Another limitation was the amount of time respondents took to complete the instrument, mainly those with lower education levels. As in previous studies on the psychometric quality of the COPSOQ III in other samples (with one exception [19]), it was not possible to include other instruments in the study to assess other types of validity, such as convergent validity. Similarly, a test-retest procedure could not be applied to assess test-retest or predictive validity.

## Conclusion

It is undeniable the importance of providing employers and those responsible for health and safety management in organizations with robust tools to identify psychosocial factors. The Portuguese middle version of COPSOQ III shown to be valid and reliable in this study. This study is part of a wider validation process of the middle version of the COPSOQ III for Portugal and is of particular importance as it provides companies, researchers and national health and safety authorities with a tool for the assessment and monitoring of psychosocial factors. Benchmark reference values are of major relevance in a perspective of occupational health.

The research and intervention experience with the middle version of COPSOQ over the last twenty-five years has shown that this instrument is very useful in various contexts, particularly in occupational health. This relevance, update and pertinence is now reinforced by this validated version, which allows not only the identification of psychosocial risk factors, but also interventions to prevent them. The Portuguese middle version of COPSOQ III provides researchers and practitioners with an essential tool for identifying, monitoring and managing psychosocial factors at company and national levels. Regularly collecting reliable information is vital for informing local and sectoral policies and strategies in psychosocial risks prevention.

## Supporting information

S1 File Portuguese middle version of COPSOQ III (COPSOQ-PT). (DOCX)

S2 File HTMT values for all possible pairs of constructs matrix. (XLSX)
