# Supplementary material for: Validation of the third version of the Copenhagen Psychosocial Questionnaire for Portugal
Source: PLOS Glob Public Health. 2026 May 19;6(5):e0006036. doi: 10.1371/journal.pgph.0006036 (PMC13186338; doi:10.1371/journal.pgph.0006036)
Supplement: S1 File — (DOCX) [file pgph.0006036.s001.docx]

Data: ___/___/_______

**VERSÃO PORTUGUESA DO QUESTIONÁRIO PSICOSSOCIAL DE COPENHAGA III (COPSOQ III – versão média)**

Cotrim, Teresa P.; Bem-Haja, Pedro; Pereira, Anabela; Fernandes, Cláudia; Azevedo, Rui; Antunes, Samuel; S. Pinto, Joaquim; Kanazawa, Flávio; Souto, Isabel; Brito, Elisabeth; Silva, Carlos F. (2022). "The Portuguese Third Version of the Copenhagen Psychosocial Questionnaire: Preliminary Validation Studies of the Middle Version among Municipal and Healthcare Workers". International Journal of Environmental Research and Public Health, 19, 3: 1167. https://doi.org/10.3390/ijerph19031167

Este questionário tem como finalidade avaliar os fatores psicossociais de risco. As perguntas estão relacionadas com a saúde, o bem-estar e os fatores do ambiente de trabalho. O questionário é sobre a sua opinião sobre as **suas** condições de trabalho. As respostas são confidenciais. Não existem respostas certas nem erradas.

Os dados recolhidos serão analisados e utilizados para fins de investigação. O tempo previsto de preenchimento do questionário é de 10 minutos. Por favor, **responda a todas as perguntas**. **Muito obrigado**.

Ao preencher este questionário assume que **conhece os objectivos do questionário e da sua utilização e aceita voluntariamente participar no estudo.**

**Parte I – Dados Sociodemográficos**

1. Sexo: ☐ Masculino ☐ Feminino
2. Idade: ________ anos
3. Setor de atividade: _____________________

**Parte II – Fatores Psicossociais**

|  | Nunca | Raramente | Às vezes | Frequen-temente | Sempre |
| --- | --- | --- | --- | --- | --- |
| 1. A sua carga de trabalho acumula-se por ser mal distribuída? | ⬜ | ⬜ | ⬜ | ⬜ | ⬜ |
| 2. Com que frequência fica com trabalho atrasado? | ⬜ | ⬜ | ⬜ | ⬜ | ⬜ |
| 3. Com que frequência não tem tempo para completar todas as tarefas do seu trabalho? | ⬜ | ⬜ | ⬜ | ⬜ | ⬜ |
| 4. Precisa de trabalhar muito rapidamente? | ⬜ | ⬜ | ⬜ | ⬜ | ⬜ |
| 5. Trabalha a um ritmo elevado ao longo de toda a jornada de trabalho? | ⬜ | ⬜ | ⬜ | ⬜ | ⬜ |
| 6. O seu trabalho exige a sua atenção constante? | ⬜ | ⬜ | ⬜ | ⬜ | ⬜ |
| 7. O seu trabalho requer que memorize muitas informações? | ⬜ | ⬜ | ⬜ | ⬜ | ⬜ |
| 8. O seu trabalho requer que seja bom a propor novas ideias? | ⬜ | ⬜ | ⬜ | ⬜ | ⬜ |
| 9. O seu trabalho exige que tome decisões difíceis? | ⬜ | ⬜ | ⬜ | ⬜ | ⬜ |
| 10. O seu trabalho coloca-o/a em situações emocionalmente perturbadoras? | ⬜ | ⬜ | ⬜ | ⬜ | ⬜ |
| 11. No seu trabalho tem de lidar com os problemas pessoais de outras pessoas? | ⬜ | ⬜ | ⬜ | ⬜ | ⬜ |
| 12. O seu trabalho exige emocionalmente de si? | ⬜ | ⬜ | ⬜ | ⬜ | ⬜ |
| 13. Tem um elevado grau de influência nas decisões sobre o seu trabalho? | ⬜ | ⬜ | ⬜ | ⬜ | ⬜ |
| 14. Pode influenciar a quantidade de trabalho que lhe compete a si? | ⬜ | ⬜ | ⬜ | ⬜ | ⬜ |
| 15. Tem alguma influência sobre o tipo de tarefas que faz? | ⬜ | ⬜ | ⬜ | ⬜ | ⬜ |
| 16. Tem alguma influência sobre o modo como faz o seu trabalho? | ⬜ | ⬜ | ⬜ | ⬜ | ⬜ |
| 17. O seu trabalho permite-lhe aprender coisas novas? | ⬜ | ⬜ | ⬜ | ⬜ | ⬜ |
|  | Nunca | Raramente | Às vezes | Frequen-temente | Sempre |
| 18. O seu trabalho permite-lhe usar as suas competências ou capacidades? | ⬜ | ⬜ | ⬜ | ⬜ | ⬜ |
| 19. O seu trabalho permite-lhe desenvolver as suas competências? | ⬜ | ⬜ | ⬜ | ⬜ | ⬜ |
| 20. Pode decidir quando faz as suas pausas? | ⬜ | ⬜ | ⬜ | ⬜ | ⬜ |
| 21. Pode tirar férias mais ou menos quando deseja? | ⬜ | ⬜ | ⬜ | ⬜ | ⬜ |
| 22. Pode fazer uma pausa no trabalho para falar com um/a colega? | ⬜ | ⬜ | ⬜ | ⬜ | ⬜ |
|  | Nada | Um pouco | Moderadamente | Muito | Extremamente |
| 23. O seu trabalho tem algum significado para si? | ⬜ | ⬜ | ⬜ | ⬜ | ⬜ |
| 24. Sente que o seu trabalho é importante? | ⬜ | ⬜ | ⬜ | ⬜ | ⬜ |
| 25. Sente-se motivado e envolvido com o seu trabalho? | ⬜ | ⬜ | ⬜ | ⬜ | ⬜ |
| 26. Gosta de falar com os outros sobre o seu local de trabalho? | ⬜ | ⬜ | ⬜ | ⬜ | ⬜ |
| 27. Sente orgulho em pertencer a esta organização / empresa? | ⬜ | ⬜ | ⬜ | ⬜ | ⬜ |
|  | Nunca | Raramente | Às vezes | Frequen-temente | Sempre |
| 28. No seu local de trabalho é informado/a com antecedência sobre decisões importantes, mudanças ou planos para o futuro? | ⬜ | ⬜ | ⬜ | ⬜ | ⬜ |
| 29. Recebe toda a informação de que necessita para fazer bem o seu trabalho? | ⬜ | ⬜ | ⬜ | ⬜ | ⬜ |
| 30. O seu trabalho é reconhecido e apreciado pela gestão de topo? | ⬜ | ⬜ | ⬜ | ⬜ | ⬜ |
| 31. A gestão de topo do seu local de trabalho respeita-o/a? | ⬜ | ⬜ | ⬜ | ⬜ | ⬜ |
| 32. É tratado/a de forma justa no seu local de trabalho? | ⬜ | ⬜ | ⬜ | ⬜ | ⬜ |
| 33. O seu trabalho tem objectivos claros? | ⬜ | ⬜ | ⬜ | ⬜ | ⬜ |
| 34. Sabe exatamente quais as suas responsabilidades? | ⬜ | ⬜ | ⬜ | ⬜ | ⬜ |
|  | Nunca | Raramente | Às vezes | Frequen-temente | Sempre |
| 35. Sabe exatamente o que é esperado de si? | ⬜ | ⬜ | ⬜ | ⬜ | ⬜ |
| 36. Faz coisas no seu trabalho com que uns concordam mas outros não? | ⬜ | ⬜ | ⬜ | ⬜ | ⬜ |
| 37. Por vezes tem que fazer coisas que deveriam ser feitas de outra maneira? | ⬜ | ⬜ | ⬜ | ⬜ | ⬜ |
| 38. Por vezes tem que fazer coisas que considera desnecessárias? | ⬜ | ⬜ | ⬜ | ⬜ | ⬜ |

| **Em relação à sua chefia direta, até que ponto considera que…** | | | | | |
| --- | --- | --- | --- | --- | --- |
|  | Nunca | Raramente | Às vezes | Frequen-temente | Sempre |
| 39. Oferece aos indivíduos e ao grupo boas oportunidades de desenvolvimento / formação? | ⬜ | ⬜ | ⬜ | ⬜ | ⬜ |
| 40. Faz um bom planeamento do trabalho? | ⬜ | ⬜ | ⬜ | ⬜ | ⬜ |
| 41. É eficaz a resolver conflitos? | ⬜ | ⬜ | ⬜ | ⬜ | ⬜ |
| 42. Dá prioridade à satisfação da equipa no trabalho? | ⬜ | ⬜ | ⬜ | ⬜ | ⬜ |

| **As questões que se seguem referem-se ao seu local de trabalho no seu todo.** | | | | | |
| --- | --- | --- | --- | --- | --- |
|  | Nunca | Raramente | Às vezes | Frequen-temente | Sempre |
| 43. Com que frequência tem ajuda e apoio dos seus colegas de trabalho, se necessário? | ⬜ | ⬜ | ⬜ | ⬜ | ⬜ |
| 44. Com que frequência os seus colegas estão receptivos a ouvi-lo/a sobre os seus problemas de trabalho, se necessário? | ⬜ | ⬜ | ⬜ | ⬜ | ⬜ |
| 45. Com que frequência os seus colegas falam consigo sobre o seu próprio desempenho laboral? | ⬜ | ⬜ | ⬜ | ⬜ | ⬜ |
| 46. Com que frequência a sua chefia direta fala consigo sobre como está a decorrer o seu trabalho? | ⬜ | ⬜ | ⬜ | ⬜ | ⬜ |
| 47. Com que frequência tem ajuda e apoio da sua chefia direta, se necessário? | ⬜ | ⬜ | ⬜ | ⬜ | ⬜ |
| 48. Com que frequência a sua chefia direta fala consigo sobre o seu desempenho laboral? | ⬜ | ⬜ | ⬜ | ⬜ | ⬜ |
| 49. Existe um bom ambiente de trabalho entre si e os seus colegas? | ⬜ | ⬜ | ⬜ | ⬜ | ⬜ |
|  |  |  |  |  |  |
|  | Nunca | Raramente | Às vezes | Frequen-temente | Sempre |
| 50. No seu local de trabalho sente-se parte de uma comunidade? | ⬜ | ⬜ | ⬜ | ⬜ | ⬜ |
| 51. Existe uma boa cooperação entre os colegas de trabalho? | ⬜ | ⬜ | ⬜ | ⬜ | ⬜ |

|  | Nada | Um pouco | Moderada-  mente | Muito | Extrema-  mente |
| --- | --- | --- | --- | --- | --- |
| 52. Sente-se preocupado/a em ficar desempregado/a? | ⬜ | ⬜ | ⬜ | ⬜ | ⬜ |
| 53. Sente-se preocupado/a com a dificuldade em encontrar outro trabalho se ficar desempregado/a? | ⬜ | ⬜ | ⬜ | ⬜ | ⬜ |
| 54. Sente-se preocupado/a em ser transferido/a para outro posto de trabalho? | ⬜ | ⬜ | ⬜ | ⬜ | ⬜ |
| 55. Preocupa-o/a que o seu horário de trabalho (turno, dias úteis, hora de entrada e saída...) seja mudado? | ⬜ | ⬜ | ⬜ | ⬜ | ⬜ |
| 56. Sente-se preocupado/a com uma diminuição na sua retribuição (redução, introdução de remuneração variável...)? | ⬜ | ⬜ | ⬜ | ⬜ | ⬜ |
| 57. Está satisfeito (a) com a qualidade do trabalho realizado por si? | ⬜ | ⬜ | ⬜ | ⬜ | ⬜ |

|  | Nunca | Raramente | Às vezes | Frequen-temente | Sempre |
| --- | --- | --- | --- | --- | --- |
| 58. Os trabalhadores confiam uns nos outros de um modo geral? | ⬜ | ⬜ | ⬜ | ⬜ | ⬜ |
| 59. Os trabalhadores ocultam informações uns dos outros? | ⬜ | ⬜ | ⬜ | ⬜ | ⬜ |
| 60. Os trabalhadores ocultam informação à gestão de topo? | ⬜ | ⬜ | ⬜ | ⬜ | ⬜ |
| 61. A gestão de topo confia nos seus trabalhadores para fazerem o seu trabalho bem? | ⬜ | ⬜ | ⬜ | ⬜ | ⬜ |
| 62. Os trabalhadores confiam na informação que lhes é transmitida pela gestão de topo ? | ⬜ | ⬜ | ⬜ | ⬜ | ⬜ |
| 63. Os trabalhadores podem expressar as suas opiniões à gestão de topo? | ⬜ | ⬜ | ⬜ | ⬜ | ⬜ |
|  |  |  |  |  |  |
|  | Nunca | Raramente | Às vezes | Frequen-temente | Sempre |
| 64. Os conflitos são resolvidos de uma forma justa? | ⬜ | ⬜ | ⬜ | ⬜ | ⬜ |
| 65. O trabalho é distribuído de forma justa? | ⬜ | ⬜ | ⬜ | ⬜ | ⬜ |
| 66. As sugestões dos trabalhadores são tratadas de forma séria pela gestão de topo? | ⬜ | ⬜ | ⬜ | ⬜ | ⬜ |
| 67. Quando os trabalhadores fazem um bom trabalho são reconhecidos? | ⬜ | ⬜ | ⬜ | ⬜ | ⬜ |

| **As próximas três questões referem-se ao modo como o seu trabalho afeta a sua vida privada/ familiar:** | | | | | |  |
| --- | --- | --- | --- | --- | --- | --- |
|  | Nunca | Raramente | Às vezes | Frequen-temente | Sempre | |
| 68. Sente que o seu trabalho lhe exige tanta energia, que acaba por afetar a sua vida privada / familiar negativamente? | ⬜ | ⬜ | ⬜ | ⬜ | ⬜ |  |
| 69. Sente que o seu trabalho lhe exige tanto tempo, que acaba por afetar a sua vida privada / familiar negativamente? | ⬜ | ⬜ | ⬜ | ⬜ | ⬜ |  |
| 70. As exigências do seu trabalho interferem com a sua vida privada e familiar? | ⬜ | ⬜ | ⬜ | ⬜ | ⬜ |  |

| **Em relação ao seu trabalho em geral, quão satisfeito(a) está com...** | | | | | | | | | | | |
| --- | --- | --- | --- | --- | --- | --- | --- | --- | --- | --- | --- |
|  | Nada | | Um pouco | | Moderadamente | | Muito | | Extremamente | | |
| 71. As suas perspetivas de trabalho? | ⬜ | | ⬜ | | ⬜ | | ⬜ | | ⬜ | | |
| 72. O seu trabalho de uma forma global? | ⬜ | | ⬜ | | ⬜ | | ⬜ | | ⬜ | | |
| 73. A forma como as suas capacidades e competências são usadas? | ⬜ | | ⬜ | | ⬜ | | ⬜ | | ⬜ | | |
|  | | Excelente | | Muito boa | | Boa | | Razoável | | Fraca |  |
| 74. Em geral, sente que a sua saúde é: | | ⬜ | | ⬜ | | ⬜ | | ⬜ | | ⬜ |  |
|  | |  | |  | |  | |  | |  |  |
|  | | Nunca | | Raramente | | Às vezes | | Frequen-temente | | Sempre |  |
| 75. Sou sempre capaz de resolver problemas se tentar o suficiente. | | ⬜ | | ⬜ | | ⬜ | | ⬜ | | ⬜ |  |
| 76. É fácil seguir os meus planos e atingir os meus objectivos. | | ⬜ | | ⬜ | | ⬜ | | ⬜ | | ⬜ |  |

| **Com que frequência durante as últimas 4 semanas …** | | | | | | | |
| --- | --- | --- | --- | --- | --- | --- | --- |
|  | Nunca | Raramente | Às vezes | Frequen-temente | Sempre | |  |
| 77. Sentiu dificuldade em adormecer? | ⬜ | ⬜ | ⬜ | ⬜ | ⬜ |  |  |
| 78. Acordou várias vezes durante a noite e depois não conseguia adormecer novamente? | ⬜ | ⬜ | ⬜ | ⬜ | ⬜ |  |  |
| 79. Tem-se sentido fisicamente exausto/a? | ⬜ | ⬜ | ⬜ | ⬜ | ⬜ |  |  |
| 80. Tem-se sentido emocionalmente exausto/a? | ⬜ | ⬜ | ⬜ | ⬜ | ⬜ |  |  |
| 81. Tem-se sentido irritado/a? | ⬜ | ⬜ | ⬜ | ⬜ | ⬜ |  |  |
| 82. Tem-se sentido ansioso/a? | ⬜ | ⬜ | ⬜ | ⬜ | ⬜ |  |  |
| 83. Tem-se sentido triste? | ⬜ | ⬜ | ⬜ | ⬜ | ⬜ |  |  |
| 84. Tem sentido falta de interesse por coisas do quotidiano? | ⬜ | ⬜ | ⬜ | ⬜ | ⬜ |  |  |
